# Congenital Cataracts and Microphakia with Retinal Dysplasia and Optic Nerve Hypoplasia in a Calf

**DOI:** 10.1155/2021/2064103

**Published:** 2021-09-06

**Authors:** Christopher L. Siepker, Jennifer L. Zimmer, Kathleen M. Bedard, Kelsey A. Hart, Sarah L. Czerwinski, K. Paige Carmichael

**Affiliations:** ^1^Iowa State University Veterinary Diagnostic Laboratory, Ames, IA, USA; ^2^Department of Small Animal Medicine & Surgery, College of Veterinary Medicine, University of Georgia, Athens, GA, USA; ^3^Department of Large Animal Medicine, Large Animal Internal Medicine Service, College of Veterinary Medicine, University of Georgia, Athens, GA, USA; ^4^Department of Pathology, College of Veterinary Medicine, University of Georgia, Athens, GA, USA

## Abstract

*Case Description*. A two-month-old, female, Aberdeen-Angus calf was presented for congenital cataracts and blindness in both eyes (OU). The dam had a reported history of visual defects (not specified) and had produced other affected calves (per owner history). Ophthalmic examination revealed mature bilateral cataracts, attenuation of the iridic granules, persistent pupillary membranes, and dyscoric pupils. Additionally, the calf had a poor body condition, prognathism, dome-shaped head, excessive nasal drainage, limb contracture, and fever. Histopathology of both eyes revealed lenticular degeneration (congenital cataracts), retinal dysplasia, and optic nerve hypoplasia. BVDV IHC detected antigen within only the left eye (OS), consisting of intrahistiocytic and endothelial immunoreactivity within the ciliary body, iris, and choroid. No BVDV immunoreactivity could be detected in the right eye (OD). This case highlights the unique ocular changes present in in utero BVDV infection of cattle with a different immunohistochemical staining profile than previously described.

## 1. Introduction

Bovine viral diarrhea virus (BVDV) is an important viral disease in cattle worldwide belonging to the genus *Pestivirus* of the family Flaviviridae similar to viruses which cause Border disease and classical swine fever [1, 2]. The disease, first described in upper New York in 1946 causes significant pathology in cattle ranging from birth defects, immunosuppression, to immunotolerant, persistently infected (PI) animals [3]. PI animals represent the major reservoir and source of infection in the herd [4]. BVDV isolates are diverse, characterized by two predominant genotypes within the United States, type 1 and 2, which are further classified into cytopathogenic (cp) and noncytopathogenic (ncp) strains, based on cell culture characteristics [5]. Diagnosis and detection of BVDV in tissues are complicated by a complex pathogenesis, resulting in variable clinical presentations and lesions [6]. Infections may occur acutely, in utero, as persistent infection (PI) or as a mucosal disease [6, 7].

In nonpregnant, immunocompetent cattle, acute infections occur with the entry of ncp BVDV into the host via the oropharyngeal mucosa, ingestion, or inhalation. Replication may begin in epithelial cells of the palatine tonsils and then spreads throughout the bloodstream. The virus can then disseminate throughout the host via attachment and entry of macrophages and lymphocytes through the CD46 receptor [7, 8]. Results of acute infection cause a wide range of effects, stemming mainly from immunosuppression, allowing for increased secondary infections, such as respiratory disease and diarrhea [9]. A majority of immunocompetent cattle will manifest with subclinical disease [10, 11].

Fetal infections occur in the pregnant dam as BVDV readily crosses the placenta, causing intrauterine infections with various manifestations, dependent on the gestation stage of the fetus. Common congenital abnormalities of fetuses infected in utero with BVDV consist of abortion, cerebellar hypoplasia, cataracts, retinal degeneration, optic neuritis, skeletal malformations, and stunted growth [12–14]. Immunotolerance in calves infected in utero, during the first trimester with a ncp strain of BVDV will result in a persistently infected (PI) calf [15]. Persistence of the virus occurs through evasion of the innate and adaptive immune responses [16]. Many mechanisms of immune evasion occur in PI cattle; however, one important method described includes failure of type I interferon secretion within ncp BVDV-infected macrophages [17].

Few case reports have been published discussing the ocular changes in cattle infected in utero with BVDV; however, ocular manifestations of disease may occur in up to 10% of calves infected with BVD [18]. Gross ocular changes have been reported to range from slight opacities of the lenses to severe microphthalmia with retinal dysplasia and detachment. Histologic findings typically observed are consistent with microphakia, persistent pupillary membranes, retinal atrophy and dysplasia with detachment, and optic nerve hypoplasia [13, 19]. BVDV antigen has previously been detected in retinal blood vessels and with multifocal staining in the outer plexiform layer of the [20].

## 2. Case Presentation

**Figure 1:**
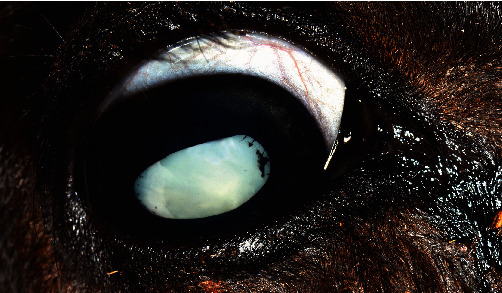
Antemortem image of the right eye showing lens opacity (cataract), persistent pupillary membranes, and lack of iridic granules.

A two-month-old, female, Aberdeen-Angus calf was referred to the University of Georgia (UGA) ophthalmology service for evaluation of congenital cataracts and blindness. The dam had a history of visual defects and previously produced affected calves (per history reported by the owner). Ophthalmic examination in the calf was limited to the anterior segment and revealed opaque lenses consistent with cataracts, attenuation of the iridic granules, persistent pupillary membranes, and dyscoric pupils (Figure 1) bilaterally, as well as the left eye had a corneal ulcer with associated off-white infiltrate. Physical examination revealed a poor body condition, prognathism, dome-shaped head, excessive nasal drainage, mild contracture of the limbs, and fever. Despite attempts at medical therapy, euthanasia was elected via pentobarbital overdose and both eyes were submitted to the UGA Diagnostic Ophthalmic Pathology Service. Each globe was placed into an individual container of 10% neutral buffered formalin for routine histology. A full necropsy of the calf was not performed.

**Figure 2:**
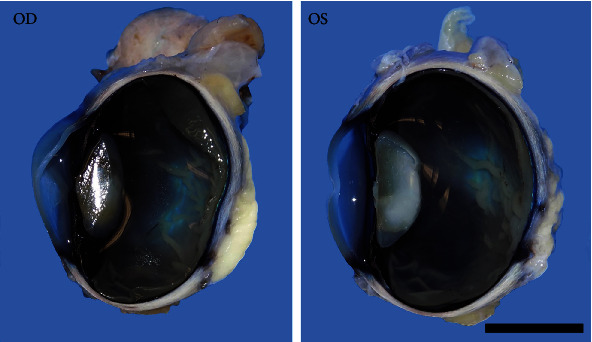
Both eyes sectioned dorsoventrally, showing a soft, gelatinous, irregular lens, and numerous small, linearized retinal folds. Both eyes were small for the reported age of the calf. Bar = 1 cm.

For the age of the calf, both globes were bilaterally and symmetrically small, measuring approximately 2.4 cm × 2.6 cm^2^. Gross findings revealed bilateral wrinkling of the ventral retina, a small optic nerve, and a small, soft, opaque, malformed lens (Figure 2). Both globes were bisected along their respective sagittal planes, dorsoventrally, and placed into individual, corresponding tissue cassettes for paraffin embedding and sectioning. All sections were stained with routine hematoxylin and eosin (H&E) and examined using light microscopy. Routine BVDV immunohistochemistry (IHC) was performed using BVDV MAb, catalog I-3.12F1 (manufactured by J. T. Saliki UGA).

**Figure 3:**
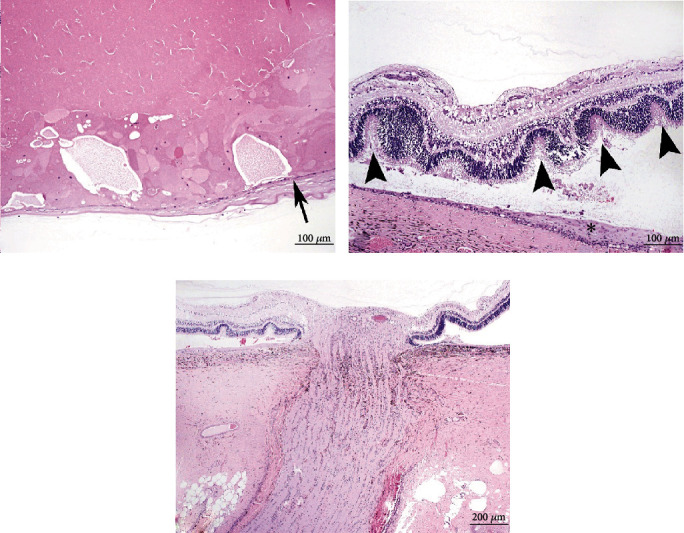
(a) Formation of Morgagnian globules and bladder cells with cystic liquefaction of the lens fibers. Posterior migration of epithelium was a prominent feature of both eyes (arrow). The lens capsule is variably wavy and irregular. Bar = 100 *μ*m. (b) Severe retinal folding and the formation of numerous rosettes with vacuolated photoreceptors (arrowheads). A spindloid population of cells replaces the RPE (asterisk). Bar = 100 *μ*m. (c) Optic nerve hypoplasia. Bar = 200 *μ*m. Routine H&E.

**Figure 4:**
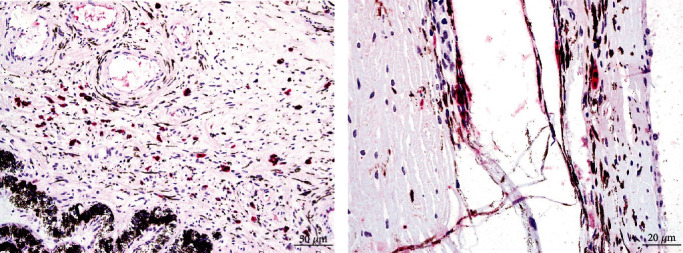
Scattered intrahistiocytic and endothelial immunoreactivity to BVDV antigen within the ciliary body and iris (a) and choroid (b). BVDV immunohistochemistry. Bars = 50 *μ*m (a) and 20 *μ*m (b), respectively.

Histologically, lesions in both eyes were similar. Severe and diffuse lenticular degeneration that was more severe OD than OS, characterized by cortical Morgagnian globules, bladder cells, and liquefaction of the nuclear fibers (cataracts). Lens epithelial cells were disorganized, variably stacked, binucleated, and spindloid with posterior migration of the epithelial layer (Figure 3(a)). The retina was extensively folded, forming multifocal rosette-like structures with detachment from the retinal pigmented epithelium (RPE). Retinal blood vessels were multifocally surrounded by a mild inflammatory infiltrate. Multifocally, replacing and apparently continuous with the retinal pigmented epithelium was a band of connective tissue composed of densely packed spindloid cells of variable thickness. The photoreceptor layer contained variably sized, discrete, clear vacuoles (Figure 3(b)). Abundant collagenous stroma replaced nerve bundles in the small optic nerve head (Figure 3(c)). Scattered, intrahistiocytic immunoreactivity to BVDV antigen was observed within the ciliary body and iris (Figure 4(a)) of the left eye. Endothelial cells exhibited scattered immunoreactivity within the choroid (Figure 4(b)). No BVDV immunoreactivity was detected in the right eye (OD).

Six scrolls of formalin-fixed paraffin-embedded tissue (FFPE) tissue from each eye submitted for BVDV type 1 and 2 multiplex polymerase chain reaction (PCR) testing were negative, thus confirmation of BVDV nucleic acid in tissue was not possible, given the samples received.

## 3. Discussion

Detection of BVDV-infected animals and especially those considered to be persistently infected, represent a significant challenge in veterinary diagnostics. To date, immunohistochemistry of haired skin represents the best tissue to detect BVDV antigen in tissues antemortem. The pathology relating to the central nervous system, immune system, and special senses is largely unknown, complicated by transient nature of disease, postinfection period, immune status, and strain of virus. Specifically, the ocular changes of cattle naturally infected with BVDV in utero are rarely reported in veterinary medicine, and the pathology outlining the potentially severe ophthalmic changes is poorly understood. This case represents a calf infected with BVDV which resulted in microphthalmia, cataracts, microphakia, and retinal dysplasia, resulting in loss of vision, clinically. While not able to entirely be confirmed without blood or ear notch sampling, the gross and histomorphic findings in both eyes are strongly suggestive of in utero BVDV infection in this calf. Gross and histologic findings correlate with previously reported ocular changes in calves infected with BVDV in utero [13, 20]. Compared to previous reports and to the authors' knowledge, this is the first reported case of congenital BVDV infection with detectable BVDV antigen noted within histiocytes and endothelium of the choroid, ciliary body, and iris [13, 20]. In contrast to previous publications, no BVDV immunoreactivity was detected within the retina or associated vasculature in this calf. The distribution of BVDV antigen observed within this calf may correlate with the advanced age status of the animal or when immunotolerance of the ncp BVDV strain occurred.

Ideally, a diagnosis of BVDV infection would include detection of BVDV nucleic acid in tissue and PCR represents a sensitive method for detecting BVDV in fresh tissues and feces [21]. The overall tissue paucity, multifocal, and unilateral distribution of BVDV immunolabeling observed may to some degree explain negative PCR results. RNA is commonly extracted from FFPE tissues in the diagnostic setting; however, issues associated with nucleic acid degradation, fragmentation, and modification may reduce PCR sensitivity [22]. The strong immunohistochemical reactivity, coupled with histomorphic changes consistent with BVDV, allowed the author's to conclude that the associated ocular defects were secondary to BVDV infection in utero.

Differential diagnoses in ruminants or cattle, based on the described gross and histologic findings, should consist of epizootic hemorrhagic disease virus (EDV) and/or blue tongue virus (BTV), hypovitaminosis A, and underlying genetic abnormalities [23]. Ideally, a full postmortem examination of the carcass would have been performed to look for additional evidence of in utero BVDV infection (i.e., cleft palate, cerebellar hypoplasia, possible limb deformities, and perifollicular immunoreactivity to BVDV in the dermis). Furthermore, follow-up testing of the dam and remaining herd mates was recommended; however, declined. No testing for EHD, BTV, or vitamin levels could be tested in the submitted tissues.

Postmortem evaluation of the globe and orbit in young calves and other small ruminants with a history of blindness or microphthalmia may be warranted and may possibly aid in the detection of persistently infected BVDV animals within the herd. Immunohistochemical detection of viral antigen within pestivral-induced ocular lesions remains a variably inconsistent method for detection of the virus.
